# Nonspecific intraventricular conduction delay predicts the prognosis of dilated cardiomyopathy

**DOI:** 10.1186/s12872-023-03437-y

**Published:** 2023-08-18

**Authors:** Yong Yuan, Kai Yang, Qianjun Liu, Weixiang Song, Dongsheng Jin, Shihua Zhao

**Affiliations:** 1 Department of Magnetic Resonance Imaging, Cardiovascular Imaging and Intervention Center, State Key Laboratory of Cardiovascular Disease, National Center for Cardiovascular Diseases, Fuwai Hospital, Chinese Academy of Medical Sciences and Peking Union Medical College, Beijing, 100037 China; 2 Department of Diagnostic Imaging, Geriatric Hospital of Nanjing Medical University, Nanjing, 210024 China; 3 Department of Cardiology, Huadong Hospital Affiliated to Fudan University, Shanghai, 200040 China; 4 Department of Radiology, Second Affiliated Hospital of Chongqing Medical University, Chongqing, 400010 China

**Keywords:** Prognosis, Dilated cardiomyopathy, Bundle branch block, Magnetic resonance imaging

## Abstract

**Purpose:**

Left bundle branch block (LBBB) has been confirmed to be independently associated with adverse outcomes in dilated cardiomyopathy (DCM). However, prognostic data on nonspecific intraventricular conduction delay (NSIVCD) are still limited and conflicting. We aimed to evaluate the prognosis of DCM with NSIVCD.

**Methods:**

A total of 548 DCM patients who underwent cardiovascular magnetic resonance imaging (CMR) from January 2016 to December 2017 were consecutively enrolled. The cohort was divided into four groups: 87 with LBBB, 27 with RBBB, 61 with NSIVCD, and 373 without intraventricular conduction delay (IVCD). After a median follow-up of 58 months (interquartile range: 47–65), 123 patients reached the composite endpoints, which included cardiovascular death, heart transplantation, and malignant arrhythmias. The associations between different patterns of IVCD and the outcomes of DCM were analysed by Kaplan‒Meier analysis and Cox proportional hazards regression analysis.

**Results:**

Of 548 DCM patients, there were 398 males (72.6%), and the average age was 46 ± 15 years, ranging from 18 to 76 years. In Kaplan‒Meier analysis, patients with NSIVCD and LBBB showed higher event rates than patients without IVCD, while RBBB patients did not. By multivariate Cox regression analysis, LBBB, NSIVCD, NYHA class, left ventricular ejection fraction (LVEF), indexed left ventricular end-diastolic diameter (LVEDDI), percentage of late gadolinium enhancement mass (LGE%), and global longitudinal strain (GLS) were found to be independently associated with the outcomes of DCM.

**Conclusions:**

In addition to LBBB, NSIVCD was an unfavourable prognostic marker in patients with DCM, independent of LVEDDI, NYHA class, LVEF, LGE%, and GLS.

## Introduction

Dilated cardiomyopathy (DCM) is the most common cause of heart failure and cardiac transplantation worldwide [1], with a reported 5-year mortality of 21–23% [2]. It is very important to identify the latent prognostic predictors of DCM. In addition to left ventricular ejection fraction (LVEF), it has been reported that arrhythmias play an important role in the outcome of DCM [3]. Left bundle branch block (LBBB) is a common arrhythmia in DCM patients and is characterized by a widened QRS complex. The results of several prior clinical trials have already indicated that LBBB is an independent prognostic factor in DCM and is associated with high mortality [4–6].

In addition to LBBB, there are two other intraventricular conduction delays (IVCDs) with widened QRS complexes, including right branch bundle block (RBBB) and nonspecific intraventricular conduction delay (NSIVCD). NSIVSD is defined as QRS duration ≥ 110 ms in adults who do not meet the criteria for LBBB or RBBB [7]. The outcome of DCM with NSIVCD has not been clarified [8]. Some studies have used a widened QRS complex duration to analyse the outcomes [9], not distinguishing NSIVCD from LBBB by a morphological pattern of the QRS complex. Some studies included NSIVCD but often in small sample sizes or subgroup analyses [10]. Therefore, further research is needed.

Previous studies have shown that patients with DCM with regional fibrosis identified by cardiovascular magnetic resonance (CMR) late gadolinium enhancement (LGE) imaging methods have adverse outcomes [11–14]. As a quantitative evaluation of myocardial fibrosis, LGE by CMR is increasingly being considered in the diagnosis and prognosis of DCM because invasive endocardial biopsy is seldom performed. Moreover, CMR myocardial strain represents an established approach in evaluating DCM and can be used to quantify myocardial deformation. This distinctive technology differs from morphology, haemodynamics, and cardiac function methods.

Therefore, we aimed to analyse DCM in our medical centre to explore whether NSIVCD is an independent prognostic factor, utilizing measurement of cardiac morphology, cardiac function, myocardial fibrosis, and myocardial deformation by CMR.

## Methods

### Study patients

This was a retrospective study approved by the Ethics Committee of Fuwai Hospital. Written informed consent was waived due to the retrospective nature of this study by the Ethics Committee of Fuwai Hospital. The study was performed in accordance with the principles of the Declaration of Helsinki.

Of 3523 patients admitted or referred to our hospital who underwent CMR on a 3.0 T Philips MRI system from January 2016 to December 2017, 852 newly diagnosed DCM patients (symptoms occurred within 2 months) were consecutively enrolled. The diagnosis of DCM was confirmed on CMR using the World Health Organization/International Society and Federation of Cardiology definition of DCM [15]. The inclusion criteria were as follows: (1) adults aged 18 years and over; (2) LVEF < 45% on CMR; and (3) left ventricle (LV) end diastolic volume > 2 standard deviations from normal according to normograms corrected by body surface area and age [16]. The exclusion criteria were as follows: (1) coronary artery disease with severe coronary artery stenosis or myocardial ischaemia indicated by perfusion imaging or an endocardial enhancement pattern of LGE on CMR. The enrolled patients must have undergone coronary angiography, coronary CT angiography, positron emission tomography, or CMR enhanced scanning in our hospital or other hospitals; otherwise, they would be excluded; (2) cardiac valve disease sufficient to cause global systolic impairment (defined as a stenosis and regurgitation rate ≥ 50%); (3) estimated glomerular filtration rate (eGFR) < 30 ml/min/1.73 m^2^; and (4) other cardiomyopathies, such as hypertrophic cardiomyopathy, hypertensive heart disease, active myocarditis, cardiac sarcoidosis, Fabry disease, peripartum cardiomyopathy, alcoholic cardiomyopathy, metabolic cardiomyopathies, and congenital heart diseases.

**Fig. 1:**
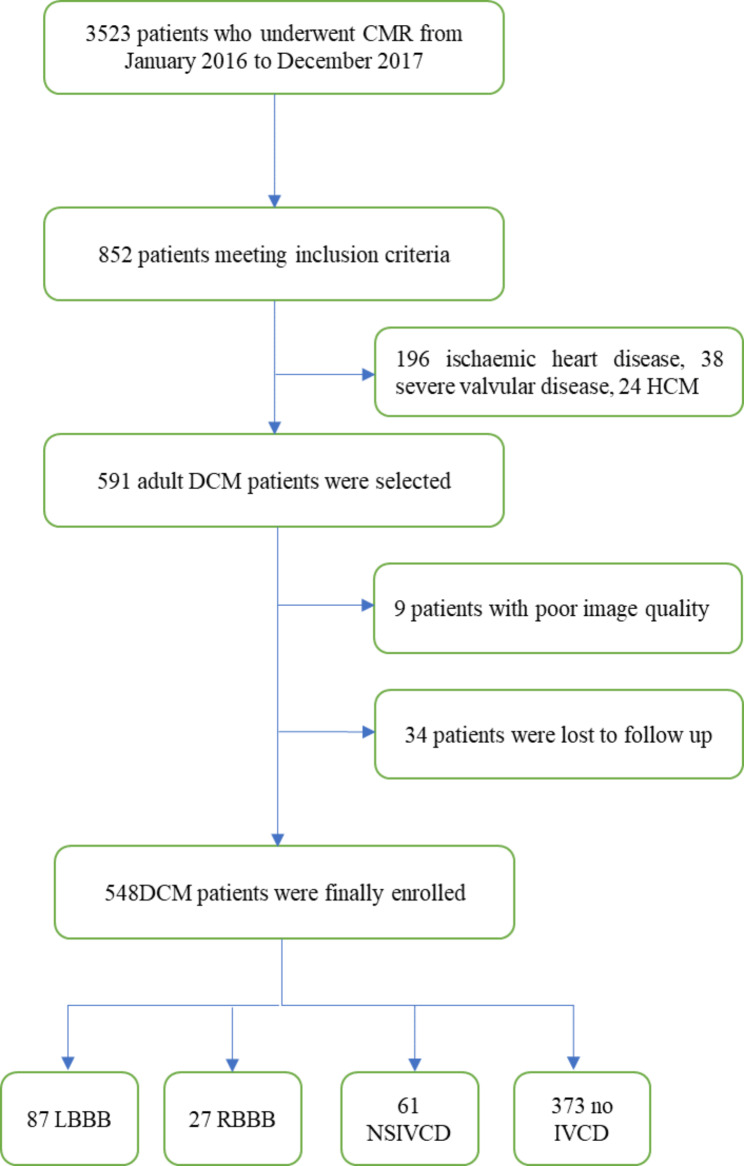
Flowchart of data acquisition, selection, and division. DCM = dilated cardiomyopathy; HCM = hypertrophic cardiomyopathy; LBBB = left bundle branch block; RBBB = right bundle branch block; NSIVCD = nonspecific intraventricular conduction delay; IVCD = intraventricular conduction delay

Of 852 DCM patients who met the inclusion criteria, 3 patients under the age of 18 were excluded; 196 cases of suspected ischaemic heart disease were excluded. Additionally, 38 cases of suspected severe valvular disease and 24 cases of suspected hypertrophic cardiomyopathy, Fabry disease, or peripartum cardiomyopathy were excluded. Then, 591 DCM patients with LVEF < 45% were selected; nine patients were excluded because of low image quality and unmatched cine phases. In addition, 34 (5.8%) patients were lost to follow-up. Finally, a total of 548 DCM patients were enrolled (Fig. 1).

By electrocardiogram, the cohort of DCM patients was divided into LBBB, RBBB, NSIVCD, and non-IVCD groups to study the prognosis. In the cohort, 87 (15.9%) had LBBB, 27 (4.9%) had RBBB, 61 (11.1%) had NSIVCD, and 373 (68.0%) had no IVCD. Diagnostic criteria for LBBB and RBBB were based on the American Heart Association/American College of Cardiology Foundation/Heart Rhythm Society recommendations [7]. The definition of LBBB was QRS duration ≥ 120 ms in adults; broad notched or slurred R wave in leads I, aVL, V5 and V6; and absent q waves in leads I, V5, and V6. The definition of RBBB was QRS duration ≥ 120 ms in adults; rsr’, rsR’, or rSR’ in leads V1 or V2; and occasionally, a wide and notched R wave and wide S waves in leads I, V5, and V6 [7, 8]. NSIVCD was defined as QRS duration ≥ 110 ms in adults who did not meet the criteria for LBBB or RBBB.

### Follow-up

The last follow-up was in December 2021. The median follow-up time was 58 months, ranging from 3 to 71 months (interquartile range: 47–65). The outcomes were composite endpoints, which included sudden cardiac death, heart failure death, heart transplantation, LV assist device installation, malignant ventricular arrhythmias, and appropriate shocks of implantable cardioverter defibrillators (ICD) or cardiac resynchronization therapy defibrillators (CRT-D) during the follow-up. Malignant ventricular arrhythmias included ventricular fibrillation, ventricular flutter, and sustained ventricular tachycardia requiring a cardioverter. All the data were obtained via medical records, clinic visits, and telephone interviews. Suspected outcomes were reviewed by two independent investigators blinded to CMR data.

### CMR protocol and analysis

#### CMR technique

All patients underwent CMR examination on a 3.0T MRI system (Philips Health care, Ingenia, Netherlands). Balanced steady-state free precision (bSSFP) cine was acquired in two-, three-, and four-chamber long-axis and short-axis views. The short-axis cine included eight slices covering the entire LV from the mitral valve ring to the apex. The main imaging parameters were as follows: slice thickness, 8 mm, no gap; repetition time, 3.4 ms; echo time, 1.5 ms; matrix size, 224 × 256; field of view, 320 × 320 mm^2^; and 25–30 phases per cardiac cycle. LGE imaging was performed 10 min after injection of contrast (0.2 mmol/kg; Magnevist; Bayer Health care, Berlin, Germany).

#### CMR analysis

The left ventricular end-diastolic diameter index (LVEDDI), left ventricular end-diastolic volume index (LVEDVI), left ventricular end-systolic volume index (LVESVI), LVEF, left atrial diameter (LAD) and left ventricular mass index (LVMI) were measured by software CVI42 (Circle Cardiovascular Imaging, Calgary, Alberta, Canada) and normalized by the body surface area calculated with the Mosteller equation. Left ventricular mass was measured at end-diastole.

**Fig. 2:**
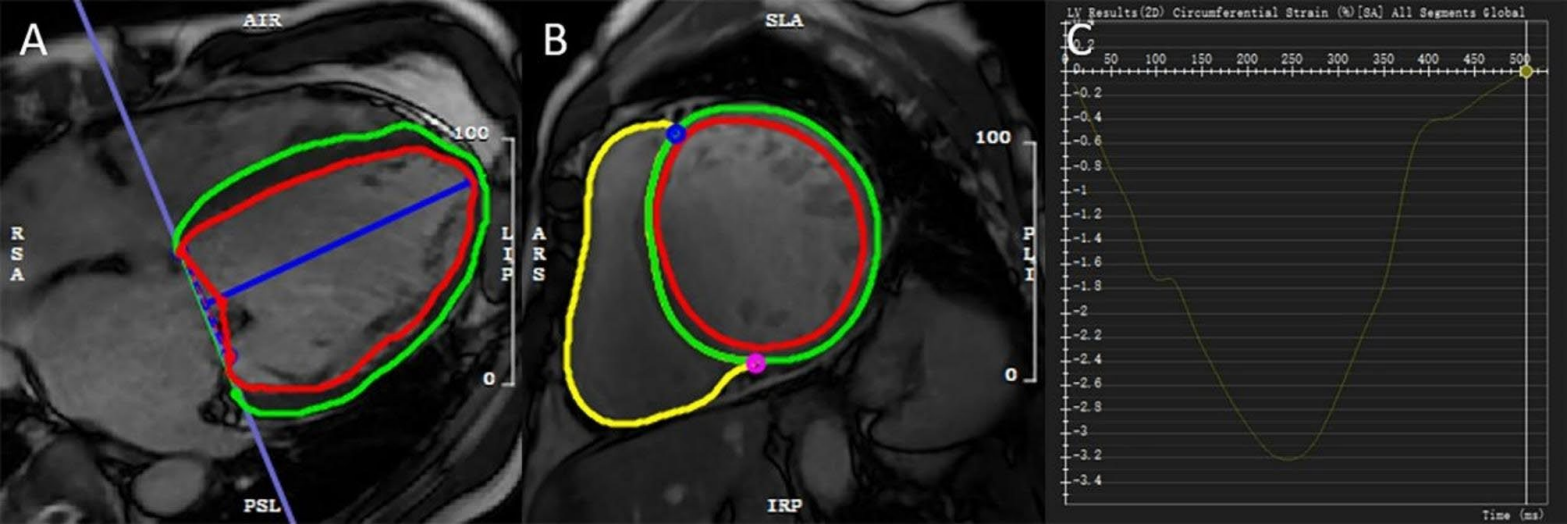
**A** and **B** show representative images of endocardial (red) and epicardial (green) contours automatically detected with manual correction in four-chamber and short-axis views. **C** shows the global circumferential strain of an NSIVCD patient. NSIVCD = nonspecific intraventricular conduction delay

CMR strain analysis by feature tracking was performed on cine images via CVI42. LV endocardial and epicardial contours were automatically tracked and manually corrected in short-axis slices and three long-axis slices in end-diastolic phases. LV global radial strain (GRS) and global circumferential strain (GCS) were derived from the short-axis cine. LV global longitudinal strain (GLS) was derived from the two-, three-, and four-chamber cine (Fig. 2).

The assessment of myocardial fibrosis was semiautomatically quantified on LGE short-axis images by the full-width half-maximum method via Qmass (Medis Medical Imaging Systems). The results were reported as a percentage of LGE mass (LGE%) to total left ventricular mass. CMR analyses were performed by a radiologist fully blinded to the clinical data.

### Intra- and interobserver agreement

Inter- and intraobserver variabilities for strain values were assessed in a randomly selected subgroup of 20 subjects with positive LGE. One observer measured the data once, and a second observer (blinded to the first observer’s results) measured the data at two time points at least two weeks apart (by Y. Y. and K. Y.).

### Statistical analysis

As appropriate, variables are presented as the means ± standard deviations, medians with interquartile ranges, or numbers with percentages. Univariate comparisons were performed by the two-sample independent t test, the Mann‒Whitney U test, one-way ANOVA, and the Pearson chi-square test for normally distributed, nonnormally distributed, and categorical variables, respectively. Kaplan‒Meier survival estimates were performed along with a log-rank test to test the proportional hazards hypothesis of Cox regression.

Univariate Cox regression was initially used to identify variables associated with the outcome. Candidate variables with a *P* value < 0.1 on univariate analysis were included in multivariate mode. Multivariate Cox regression was performed to determine independent associations with the outcomes. The results were expressed as adjusted hazard ratios (HRs) and 95% confidence intervals (CIs).

We analysed latent predictors of outcomes from several perspectives: structure remodelling, LV dysfunction, IVCD patterns, myocardial fibrosis, and LV myocardial global strain. Baseline variables considered clinically relevant to outcomes were entered into univariate Cox regression. Collinearity analysis was performed to obtain more appropriate variables by linear regression, and a variance inflation factor > 10 was considered collinear. Given the number of endpoint events, multivariate models were limited to no more than twelve parameters, allowing for approximately one covariable per 10 events to ensure parsimony of the final model. Inter- and intraobserver variabilities were analysed by the intraclass correlation coefficient (ICC). *P <* 0.05 was considered statistically significant. All analyses were performed by IBM SPSS Statistics (version 26).

## Results

### Population and follow-up

As shown in Tables 1 and 2, the average age in the cohort was 46 ± 15 years, ranging from 18 to 76 years, and there were 398 males (72.6%). The average LVEF was 29.6 ± 8.6, with 264 (48.2%) patients with LVEF ≤ 35%. A total of 123 (22.4%) patients reached the composite endpoints (cumulative event rate: 22.4%): 44 (8.0%), death from heart failure; 19 (3.5%), sudden cardiac death; 43 (7.8%), heart transplantation; 2 (0.3%), LV assist device installation; 11 (2.0%), malignant ventricular arrhythmias. During the follow-up, 24 (4.4%) patients were implanted with ICD devices, and 17 (3.1%) were implanted with CRT-D devices. Among them, 5 patients had appropriate shocks, but one patient died of heart failure. The components of clinical outcomes categorized by IVCD are summarized in Table 3.

**Table 1:** Baseline patient characteristics and CMR findings

		Composite Endpoints		
	Patients	Negative	Positive	
Variable	N = 548	N = 425	N = 123	*P* value
Demographics				
Men/women	398/150 (72.6%/27.4%)	303/122 (71.3%/28.7%)	95/28 (77.2%/22.8%)	0.193
Age (year)	46 ± 15	45 ± 12	47 ± 13	0.223
Body mass index (kg/m^2^)	25.1 ± 4.6	25.2 ± 4.4	24.8 ± 4.7	0.168
Alcohol	152 (27.7%)	118(27.8%)	34 (27.6%)	0.979
History of smoking	203 (37.0%)	151 (35.5%)	52 (42.3%)	0.172
Family history of DCM	87 (15.9%)	59 (13.9%)	28 (22.8%)	0.125
Comorbidity				
Hypertension	126 (23.0%)	91 (21.4%)	35 (28.5%)	0.102
Diabetes	96 (17.5%)	75 (17.6%)	21 (17.1%)	0.883
Hyperlipidaemia	122 (22.3%)	97 (22.8%)	25 (20.3%)	0.558
NYHA classes				< 0.001
I	35 (6.4%)	34 (8.0%)	1 (0.8%)	
II	191 (34.9%)	172 (40.5%)	19 (15.4%)	
III	218 (39.8%)	166 (39.1%)	52 (42.3%)	
IV	104 (19.0%)	53 (12.5%)	51 (41.5%)	
NT-proBNP (pg/mL)	1754 (1176–3589)	1636 (1097–3228)	1825 (1251–4659)	0.140
Electrocardiogram				< 0.001
LBBB	87 (15.9%)	53 (12.5%)	34 (27.6%)	
RBBB	27 (4.9%)	22 (5.2%)	5 (4.1%)	
NSIVCD	61 (11.1%)	28 (6.6%)	33 (26.8%)	
No IVCD	373 (68.0%)	322 (75.8%)	51 (41.5%)	
Medications				
ACE inhibitor	333 (60.8%)	255 (60.0%)	78 (63.4%)	0.495
ARB	159 (29.0%)	127 (29.9%)	32 (26.1%)	0.405
β-Blocker	481 (87.8%)	369 (86.8%)	112 (91.1%)	0.207
Diuretic MRA Digoxin	462 (84.3%) 302 (55.1%) 378 (69.0%)	357 (84.0%) 228 (53.6%) 289 (68.0%)	105 (85.4%) 74 (60.2%) 89 (72.4%)	0.714 0.211 0.358
CMR findings				
LVEDVI (ml/m^2^)	170.6 ± 56.4	161.3 ± 49.5	207.5 ± 68.3	< 0.001
LVESVI (ml/m^2^)	138.1 ± 50.6	126.7 ± 40.9	171.3 ± 61.4	< 0.001
LVEDDI (mm/m^2^)	40.6 ± 6.1	39.2 ± 5.4	43.4 ± 7.5	< 0.001
LAD (mm)	41.1 ± 9.8	40.1 ± 8.6	44.7 ± 10.7	0.033
LV mass index (g/m^2^)	85.4 ± 40.4	83.5 ± 38.3	88.1 ± 46.2	0.382
LVEF (%)	29.6 ± 8.6	36.7 ± 8.4	25.1 ± 8.5	< 0.001
Percentage of LGE (%)	5.3 (4.6–10.2)	5.0 (3.9–7.4)	9.2 (5.2–15.6)	< 0.001
GRS (%)	8.1 ± 3.2	8.4 ± 3.2	6.4 ± 2.9	< 0.001
GCS (%)	-6.1 ± 2.3	-6.5 ± 2.4	-5.3 ± 2.2	< 0.001
GLS (%)	-6.3 ± 2.4	-6.4 ± 2.5	-5.2 ± 2.5	< 0.001

Values are n (%), means ± standard deviations, or means (interquartile ranges).

ACE = angiotensin-converting enzyme; ARB = angiotensin II receptor blockade; BMI = body mass index; DCM = dilated cardiomyopathy; GCS = global circumferential strain; GLS = global longitudinal strain; GRS = global radial strain; IVCD = intraventricular conduction delay; LAD = left atrial diameter; LBBB = left bundle branch block; LGE = late gadolinium enhancement; LVEDDI = left ventricular end-diastolic diameter index; LVEDVI = left ventricular end-diastolic volume index; LVEF = left ventricular ejection fraction; LVESVI = left ventricular end-systolic volume index. MRA = mineralocorticoid receptor antagonist; NSIVCD = nonspecific intraventricular conduction delay; NYHA = New York Heart Association; RBBB = right bundle branch block.

**Table 2:** Baseline patient characteristics categorized by IVCD patterns

	LBBB N = 87	RBBB N = 27	NSIVCD N = 61	No IVCD N = 373	*P* value
Variable					
Demographics					
men/women	59/28 (67.8%/32.2%)	20/7 (74.1%/25.9%)	51/10 (83.6%/19.2%)	268/105 (71.8%/28.2%)	0.183
Age (year)	47 ± 14	45 ± 16	48 ± 14	44 ± 15	0.523
Body mass index (kg/m^2^)	25.3 ± 4.4	25.2 ± 4.5	24.8 ± 4.7	25.3 ± 4.5	0.238
Alcohol	27 (31.0%)	9 (33.3%)	16 (26.2%)	100 (26.8%)	0.001
History of smoking	34 (39.1%)	16 (59.3%)	27 (44.3%)	126 (33.8%)	0.001
Family history of DCM	19 (21.8%)	5 (18.5%)	10 (16.4%)	53 (14.2%)	0.013
Comorbidity					
Hypertension	23 (27.1%)	3 (11.5%)	14 (23.9%)	86 (27.1%)	0.106
Diabetes	14 (17.3%)	5 (18.5%)	9 (15.8%)	68 (21.1%)	0.189
Hyperlipidaemia	19 (32.2%)	5 (23.8%)	32 (31.9%)	153 (35.2%)	0.735
NYHA classes III and IV	63 (72.4%)	17 (63.0%)	45 (73.8%)	197 (52.8%)	0.001
NT-proBNP (pg/mL)	1883 (1348–3925)	2148 (1078–3764)	1722 (1269–3665)	1768 (1048–3534)	0.143
Medications					
ACE inhibitor	61 (71.8%)	12 (44.4%)	72 (70.0%)	218 (60.4%)	0.029
ARB	21 (24.4%)	9 (33.3%)	12 (20.0%)	117 (32.2%)	0.162
β-Blocker	76 (89.4%)	21 (77.8%)	54 (91.5%)	330 (91.4%)	0.132
Diuretic	73 (85.9%)	21 (77.8%)	50 (84.7%)	318 (87.1%)	0.576
MRA	47 (54.0%)	13 (48.1%)	35 (57.4%)	207 (55.5%)	0.134
Digoxin	62 (81.6%)	16 (76.2%)	49 (79.0%)	251 (85.4%)	0.448

Values are n (%), means ± standard deviations, or means (interquartile ranges).

Abbreviations are as in Table 1

**Table 3:** The components of clinical outcomes categorized by IVCD

	LBBB N = 87	RBBB N = 27	NSIVCD N = 61	No IVCD N = 373	Total
Composite endpoints	33 (37.9%)	5 (18.5%)	30 (49.2%)	55 (14.7%)	123 (22.4%)
Death from heart failure	15	3	13	23	44 (8.0%)
Sudden cardiac death	6		4	9	19 (3.5%)
Heart transplantation	9	2	13	19	43 (7.8%)
LV assist device			1	1	2 (0.3%)
Malignant ventricular arrhythmias	5		3	3	11 (2.0%)
Appropriate shocks of ICD or CRT-D	1		1	2	4 (0.7%)
Death from other causes	1	1	3	7	12 (2.2%)

CRT-D = cardiac resynchronization therapy defibrillator; ICD = implantable cardioverter defibrillator; LV = left ventricle

There were no significant differences in the sex or age distribution between the positive and negative endpoint groups. Of the 548 patients, 87 (15.9%) had a family history of DCM, including 59 (13.9%) in the negative endpoint group and 28 (22.8%) in the positive endpoint group. There was no significant difference in the proportion of family history between the negative and positive endpoint groups. There was a significant difference in prognosis between different patterns of IVCD (*P* < 0.001). Patients with New York Heart Association (NYHA) functional classes III and IV and higher NT-proBNP or troponin levels were more likely to reach the endpoints as expected. The histories of smoking and alcohol, the medications of angiotensin-converting enzyme inhibitors, angiotensin receptor blockers, β-blockers, diuretics, mineralocorticoid receptor antagonists, and digoxin between the positive and negative endpoint groups were not significantly different. Among the cohort of DCM patients divided by IVCD patterns, there were significant differences with regard to smoking and alcohol consumption. The IVCD group had a higher proportion of patients with a family history of DCM and higher NYHA classes. There were no significant differences related to medications except for ACE inhibitors. The positive endpoint group had a higher frequency of NYHA classes III and IV. The detailed baseline characteristics are summarized in Tables 1 and 2.

### Statistical analysis

Apart from the LV mass index, CMR parameters such as LVEDVI, LVESVI, LVEDDI, LVEF, LGE%, and variable strain were statistically significant between the positive and negative endpoint groups in the two-sample independent t test, Mann‒Whitney U test, and Pearson chi-square test. Compared with the negative group, patients in the positive group showed larger left ventricles, worse left ventricular ejection function, more left ventricular myocardial fibrosis, and more severe impairment of left ventricular global strain as shown in Table 1.

**Fig. 3:**
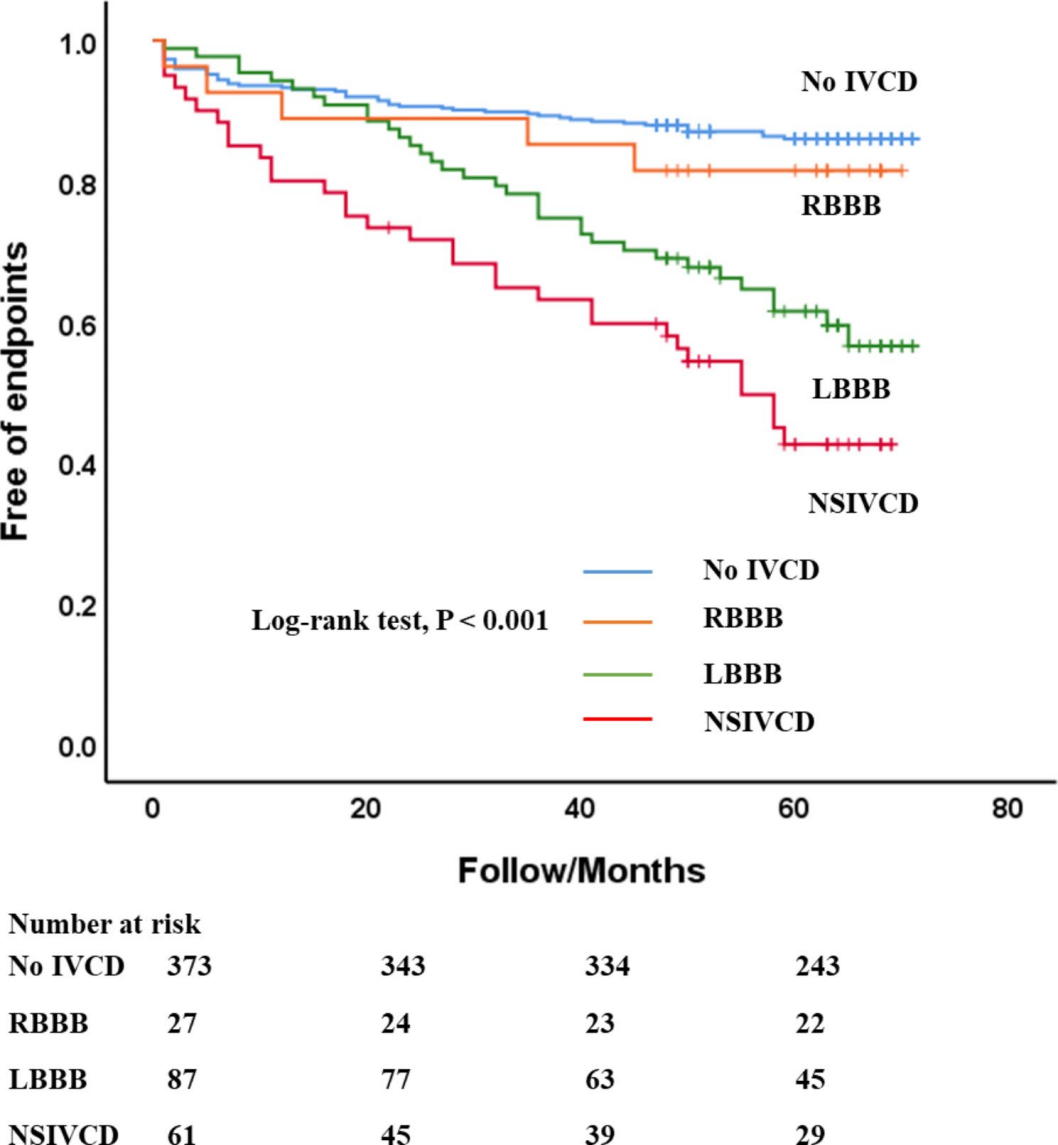
The Kaplan‒Meier plot shows the relationships of three IVCD patterns with endpoints. There were significant differences when comparing all 4 groups simultaneously (log-rank test, *P* < 0.001). IVCD = intraventricular conduction delay; NSIVCD = nonspecific intraventricular conduction delay; LBBB = left bundle branch block; RBBB = right bundle branch block

Baseline variables clinically relevant to outcomes such as sex, age, alcohol, smoking, family history of DCM, NYHA functional classes and NT-proBNP, structure remodelling parameters, LVEF, IVCD patterns, LGE%, global strain GRS, GCS, and GLS were entered into univariate Cox regression. Univariate Cox regression analysis showed that LVEDDI, LVESVI, LVEDVI, LAD, LVEF, NYHA functional classes, LGE%, IVCD patterns, and strain variables GRS, GCS, and GLS were all significantly associated with the endpoints. A collinearity analysis was performed among the remodelling variables LVEDVI, LVESVI, and LVEDDI. The variance inflation factors of LVEDVI and LVESVI were both > 10 as shown in Table 4. Therefore, only LVEDDI was selected for the multivariate Cox regression model. The set of IVCD patterns was divided according to four dummy variables: LBBB, RBBB, NSIVCD, and no IVCD. The Kaplan‒Meier curve for IVCD patterns is presented in Fig. 3. There were significant differences when comparing all 4 groups simultaneously (log-rank test, *P* < 0.001). Subsequently, we conducted pairwise Kaplan‒Meier analyses of the above four groups. Patients with NSIVCD showed higher event rates than patients without IVCD (log-rank test, *P* < 0.001). Patients with LBBB showed higher event rates than patients without IVCD (log-rank test, *P* = 0.006). There was no statistical significance between the event rates of patients with RBBB and those without IVCD (log-rank test, *P* = 0.555). There was also a difference between LBBB patients and NSIVCD patients (log-rank test, *P* = 0.039).

**Table 4:** Collinearity Analysis

Variable	Tolerance	VIF
LVEDVI	0.072	13.835
LVESVI	0.056	17.965
LVEDDI	0.520	1.924
LAD	0.435	2.688
LVEF	0.358	2.790
Percentage of LGE	0.919	1.088
LBBB	0.888	1.126
GRS	0.207	4.820
GCS	0.211	4.745
GLS	0.616	1.624

VIF = variance inflation factor

We finally selected the remodelling variables LVEDDI, LAD, cardiac function variable LVEF, NYHA functional classes, fibrosis variable LGE%, IVCD patterns, and strain variables GLS, GRS, and GCS as covariables to create the final multivariate Cox model. With stepwise analyses, LBBB (HR: 2.025; 95% CI: 1.254–3.388), NSIVCD (HR: 1.716; 95% CI: 1.092–2.854), LVEDDI (HR: 1.065; 95% CI: 1.023–1.084), LGE% (HR: 2.632; 95% CI: 1.801–3.845), LVEF (HR: 0.954; 95% CI: 0.910–0.976), and NYHA functional classes (HR: 2.132; 95% CI: 1.603–2.836) were shown to be independently associated with the endpoints after adjustment for other confounding variables (Table 5).

**Table 5:** Factors associated with adverse clinical composite outcomes

	Univariate Analysis		Multivariate Analysis		
variable	Unadjusted Hazard Ratio	*P* Value	Adjusted Hazard Ratio	*P* Value	
Sex	0.930 (0.582–1.488)	0.763			
Age (years)	1.003 (0.989–1.018)	0.631			
Hypertension	1.568 (0.731–3.019)	0.227			
Alcohol	1.354 (0.869–2.167)	0.145			
History of smoking	1.048 (0.957–1.238)	0.382			
Family history of DCM	2.462 (0.497–3.718)	0.135			
NYHA classes	2.856 (2.189–3.726)	< 0.001	2.132 (1.603–2.836)	< 0.001	
NT-proBNP (pg/mL)	1.962 (0.812–3.254)	0.342			
IVCD patterns					
LBBB	2.112 (1.300-3.433)	0.003	2.025 (1.254–3.388)	0.006	
RBBB	1.600 (0.639–4.011)	0.316	1.264 (0.501–3.185)	0.483	
NSIVCD	3.968 (2.469–6.376)	< 0.001	1.716 (1.092–2.854)	< 0.001	
LVEDDI (mm/m^2^)	1.011 (1.038–1.145)	0.003	1.065 (1.023–1.084)	0.001	
LAD (mm)	1.036 (1.004–1.069)	0.026	1.003 (0.966–1.041)	0.889	
LVEF (%)	0.921 (0.881–0.949)	< 0.001	0.954 (0.910–0.976)	< 0.001	
LGE % (%)	3.458 (2.441–5.032)	< 0.001	2.632 (1.801–3.845)	< 0.001	
GRS (%)	2.647 (1.811,3.941)	< 0.001	1.116 (0.643–1.936)	0.390	
GCS (%)	2.752 (1.853–4.505)	< 0.001	1.029 (0.385–2.756)	0.255	
GLS (%)	2.583 (1.775–3.694)	< 0.001	1.180 (0.740–1.880)	0.027	

Data in parentheses are 95% confidence intervals. Abbreviations are as in Table 1

### Reproducibility assessment

Both intra- and interobserver reproducibility were good for LV strain variables. The results of the ICC analyses are summarized in Table 6.

**Table 6:** **Intra- and interobserver variability for strain measurements**

	Intraobserver n = 20		Interobserver n = 20	
Variable	ICC	95% CI	ICC	95% CI
GRS	0.886	0.698–0.945	0.856	0.687–0.945
GCS	0.859	0.675–0.918	0.847	0.694–0.936
GLS	0.857	0.712–0.921	0.868	0.691–0.897

ICC = intraclass correlation coefficient; CI = confidence interval. Other abbreviations are the same as in Table 1

## Discussion

In this study, we explored the prognostic value of IVCD in DCM patients and found that NSIVCD was independently associated with adverse outcomes after adjusting for LVEDDI, NYHA class, LVEF, LGE%, GLS and LBBB. The Kaplan‒Meier survival curves associated with different IVCD patterns and no IVCD gradually separated over time, with a significantly lower survival rate in the NSIVCD and LBBB groups.

It is not surprising that LVEF, LVEDDI, LGE, and GLS were associated with the prognosis of DCM as LVEF is a direct factor reflecting cardiac systolic function, LVEDDI is a variable of left ventricular remodelling, and LGE represents myocardial fibrosis. In addition, GLS has been shown to be incremental to common clinical and CMR risk factors, including LVEF and LGE [17].

NT-proBNP is an indicator for evaluating heart failure, but it was found to be unrelated to prognosis, possibly due to inconsistent measurement times. Some measurements might occur during acute onset of heart failure, while others might occur during outpatient follow-up. Due to the characteristics of retrospective studies, it is difficult to measure at a fixed time.

As we mentioned in the introduction, IVCD is a series of intraventricular conduction abnormalities characterized by a widened QRS complex, including LBBB, RBBB, and NSIVCD. NSIVCD is defined as QRS duration ≥ 110 ms in adults who do not meet the criteria for LBBB or RBBB. LBBB is already found to be a critical prognostic factor of DCM. However, most investigations have focused on LBBB, so contemporary studies on the prognosis of NSIVCD are scarce. Some studies used a widened QRS complex duration to analyse the outcomes [9], not distinguishing NSIVCD from IVCD by a morphological pattern of the QRS complex. Some studies included NSIVCD but often in small sample sizes or subgroup analyses [10]. In the present study, we emphasized NSIVCD in DCM with a relatively large sample size. There are two studies on the prognosis of NSIVCD, which were based on the general population [18, 19]. They found that NSIVCD was independently associated with cardiovascular mortality in the general population. Our study is based on patients with primary DCM, which is different from the general population and nonischaemic cardiomyopathy, making the results more clinically significant for patient treatment.

One study [20] on the prognosis of DCM with IVCD in 2016 showed that NSIVCD was related to prognosis, but the researchers also concluded that RBBB was independently associated with prognosis, while LBBB was not, which was inconsistent with the mainstream viewpoint, that is, LBBB had poor prognosis and could benefit from CRT [21]. Our study provides a reference for this controversy, finding that both NSIVCD and LBBB are independently associated with adverse outcomes. When there is no LBBB on the electrocardiogram of DCM patients but there is NSIVCD, it can be determined that the prognosis may be poor, and timely and effective intervention is warranted.

LBBB induces left ventricular systolic dyssynchrony and impairs left ventricular systolic and diastolic function. Our study showed that compared to DCM patients without IVCD, LVEF was reduced in NSIVCD patients, and there were also significant differences in NYHA classes and GLS deformation parameters between patients with NSIVCD and without IVCD. Because NSIVCD is also a type of electrical dyssynchrony, similar to LBBB, mechanical dyssynchrony presented in LBBB may exist in NSIVCD patients, although mechanical dyssynchrony is not seen with simple eyeballing. Accordingly, NSIVCD may also affect DCM prognosis by affecting left ventricular systolic function. It is worth discussing whether NSIVCD patients will benefit from CRT and become potential CRT patients. Further studies are warranted to explore whether there is left ventricular systolic dyssynchrony in DCM patients with NSIVCD, similar to LBBB.

When analysing the prognosis in cases of LBBB and NSIVCD, we also analysed patients with RBBB and found that RBBB had no prognostic significance in DCM patients. Although some studies found that RBBB was independently related to the adverse prognosis of DCM [22, 23], CRT studies on IVCD showed that patients with RBBB did not benefit from CRT. However, the number of RBBB cases in our cohort was only 27, accounting for only 4.9%, which reduced the reliability of this result. In other studies on DCM with RBBB, the number of RBBB cases was also relatively small [24, 25]. RBBB, which represents the damage and fibrosis of the right ventricular myocardium, may not significantly affect the quality of life of DCM patients. Therefore, there are fewer DCM inpatients with RBBB. Like the few RBBB patients in the general population, they have few symptoms.

### Limitations

Some limitations in our study should be acknowledged. This was a hospital-based and retrospective study. The cohort was a selected population of patients who had been referred for treatment. Data bias induced by missing visits for different reasons was also inevitable. The onset time of DCM is challenging to determine in a retrospective study, which may have influenced the prognosis of DCM. The study pool was relatively young (46 ± 15 years), which may be related to genetic factors, with DCM as high as 20–35% [26]. In addition, CMR can assess LV mechanical dyssynchrony and seems to be useful to explain why LBBB and NSIVCD showed the same prognosis. Therefore, one of the limitations is that mechanical dyssynchrony was not assessed by CMR in this study.

## Conclusions

In addition to LBBB, NSIVCD was an unfavourable prognostic marker in patients with DCM, independent of LVEDDI, NYHA classes, LVEF, LGE%, and GLS. These data may help clinicians adopt appropriate treatment strategies for DCM patients with NSIVCD. Further studies are needed to explore whether there is left ventricular systolic dyssynchrony in DCM patients with NSIVCD as in those with LBBB.

## Data Availability

The datasets used and/or analysed during the current study are available from the corresponding author on reasonable request.
